# Assessing the feasibility of interactions within a computer-generated virtual reality for people with dementia

**DOI:** 10.1177/20556683251393992

**Published:** 2025-11-27

**Authors:** Alexander Prinz, Dan Bürger, Marvin Schmidt, Richard Jesse, Kerstin Witte

**Affiliations:** 1 Department of Sports Engineering/Movement Science, Otto-von-Guericke-University Magdeburg, Germany

**Keywords:** virtual reality, dementia, interaction, reaction tests, computer-generated

## Abstract

**Background:**

The global prevalence of dementia is increasing, necessitating effective non-pharmacological interventions due to the limitations and side effects of pharmacological treatments. Digital health interventions, including Virtual Reality (VR), offer promising alternatives. This study assessed the feasibility, safety, and potential side effects of interactive computer-generated VR (CGVR) for individuals with dementia (IwD), focusing on reaction tests of varying complexity.

**Methods:**

In the feasibility study recruited 32 participants with mild to moderate dementia, assessed by the Mini-Mental-State-Examination (MMSE). Participants underwent a single CGVR session involving virtual reaction wall tasks of varying difficulty. Pre- and post-exposure assessments, alongside feedback forms, evaluated cognitive and motor functions, mood, anxiety, and balance (paired t-test).

**Results:**

Of the 32 participants, 29 completed the study (9% drop-out). Observational data indicated high levels of engagement and enjoyment. No adverse effects on cognitive or motor functions were observed, with slight non-significant improvements across most parameters. MMSE scores correlated significantly with task feasibility and reaction/motor times, highlighting the influence of cognitive status on performance.

**Conclusions:**

CGVR demonstrated high feasibility and acceptability among IwD, with no adverse effects and potential to enhance cognitive and motor skills. Further research is required to explore long-term efficacy and optimize therapeutic applications.

## Background

The global prevalence of individuals with dementia (IwD) is projected to exceed 152 million by 2050.^1^ Dementia is a rapidly progressive and currently incurable neurodegenerative brain disorder that imposes a substantial societal burden, both in terms of financial costs and care requirements.^2^ Traditional treatments primarily focus on symptom reduction through pharmacological interventions, which are frequently associated with side effects.^3^ Consequently, non-pharmacological therapeutic approaches are gaining increasing importance.^4^

Physical activity interventions have shown promising effects in improving cognition and physical function, or in slowing disease-related decline.^5–7^ Furthermore, physical activity has been associated with improvements in quality of life and activities of daily living (ADL).^8–12^ At the same time, contradictory results indicate that the effects of physical activity on symptoms in IwD require further investigation.^2^ Given the heterogeneous symptomatology of dementia, effective care demands individualized interventions^13^ – something that conventional exercise programs can only partially achieve.^13^

Digital solutions provide new opportunities in this context.^14^ In recent years, such applications have increasingly been explored in the care and therapy of IwD.^14^ Digital health applications, including exergaming, video games, telemedicine, and virtual reality (VR), open up new possibilities for personalized approaches.^15,16^ However, the integration of novel technologies such as VR into dementia care remains a matter of debate.^17,18^

VR is a portable system that can flexibly address the individual limitations of IwD—adaptations that are often difficult or impossible to achieve in the real world.^19^ A distinction is made between non-immersive and immersive VR. Immersion is generated through technical specifications (e.g., wide field of view, high resolution), interactions with the virtual environment, and substantial isolation from the outside world, typically achieved via head-mounted displays (HMDs).^20^ In this study, the term VR refers exclusively to immersive VR (iVR).

The scientific literature on iVR in IwD remains limited. Most existing studies have examined the feasibility and effects of VR in older adults with cognitive or physical impairments,^21^ while only a few have specifically investigated IwD. A review by^22^ reported three studies showing high tolerance and engagement of IwD with VR. Another investigation found that IwD tolerated 360° video VR well, experienced no adverse effects, and showed interest.^23^ However, evidence that iVR could improve motor and cognitive functions or reduce fall-related anxiety is still scarce.^21,24^ A recent review by Rodriguez-Mansilla et al. (2025) confirmed this, noting that only one of the included studies actually applied iVR with IwD.^25^ Nevertheless, a meta-analysis by^26^ concluded that iVR interventions may represent promising non-pharmacological approaches to enhancing cognitive and motor functions in IwD.^26^

These findings highlight a substantial research deficit concerning the application of iVR in IwD.^21,22^ Due to small sample sizes and limited numbers of studies, no evidence-based body of research can yet be established. Moreover, many studies rely on 360° video VR—a dated technology with reduced resolution and lacking interactivity. Even iVR applications often face limitations in terms of individualization, as they are frequently implemented as stand-alone solutions. Computer-generated virtual reality environments (CGVR) may provide a way forward by enabling both higher interactivity and greater adaptability to individual needs.^27^

The use of CGVR in IwD is largely unexplored.^25,27^ Supported by advanced 3D modeling software (e.g., Blender) and game engines (e.g., Unity, Unreal Engine), CGVR offers substantial potential for interactive and individualized training and therapy scenarios. Initial studies point in this direction: for example,^28^ reported on the feasibility and positive reception of a CGVR-based intervention among IwD, while^29^ found that immersive CGVR reminiscence therapy improved mood and cognition. Previous studies have also demonstrated that a CGVR approach is feasible and may potentially enhance user interaction.^30–34^ However, these studies were conducted with relatively small sample sizes (<10),^30–34^ and only a few of them investigated more complex motor movements.^29,31,34^ While these studies underscore the potential of CGVR, they also emphasize the considerable research deficit concerning interactive, individualized CGVR interventions tailored specifically for people with dementia. Against this background, the present feasibility study aimed to evaluate the implementation, safety, and acceptance of a newly developed interactive, movement-based CGVR application for IwD, while also identifying potential adverse effects on emotional, cognitive, and motor functions.

The primary objective was to assess the feasibility of CGVR use in IwD, focusing on acceptance, safety, and interaction with the virtual environment. A secondary objective was to examine possible adverse effects of CGVR on motor and cognitive functions as well as overall well-being. In line with the existing literature, no significant deterioration is expected, nor are substantial improvements anticipated following a single exposure.

## Methods

### Study design and sample

A pre-post-design was employed to examine possible side effects of CGVR by comparing motor and cognitive functions of IwD shortly before (pretest) and directly after (posttest) interacting with CGVR. The interaction involved reaction tests with different difficulties (modes: easy, medium, hard) on a virtual reaction wall with different sizes (small: 3x3 (rows x columns) stimuli, medium: 4x3 stimuli and large: 4x5 stimuli). Different difficulty levels and wall sizes were selected to rapidly adjust task demands to the individual capabilities of the IwD, highlighting a key advantage of CGVR.

Data collection occurred in a single session lasting approximately 60 min. Power analysis with G*Power 3 (version 3.1.9.7, t-test matched pairs, α = 0.05, 1-β = 0.80, d = 0.5) resulted in a total sample size of 27 participants. Drawing on evidence from prior intervention studies, an anticipated drop-out rate in the range of 15–30% is assumed.^35,36^ However, given that the present study investigates feasibility within a single-session design without an active intervention, the lower bound of this range is considered most appropriate.^36^ Thus, 32 participants for the total sample were required. Initially, Before the pre-test, participants and their legal guardians provided written informed consent after being briefed on the study’s details.

**Figure 1.**
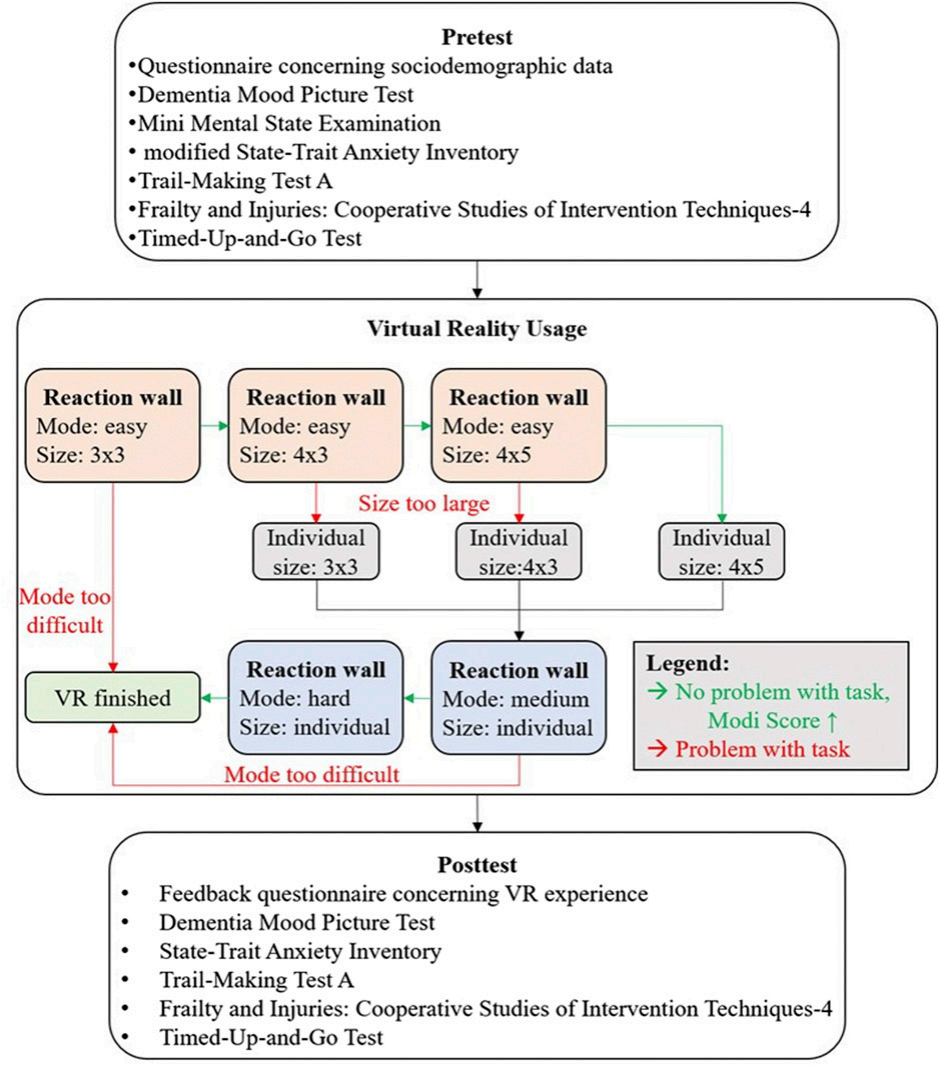
Study’s design and procedure to identify the best fitting wall size.

Recruitment involved contacting four local nursing homes that pre-selected potential participants based on predefined inclusion and exclusion criteria. Inclusion criteria encompassed being over 70 years old and having mild to moderate dementia, indicated by the Mini-Mental State Examination,^37^ while exclusion criteria were untreated hypertension, severe cardiovascular diseases, and considerable motor impairments. The study’s design is visualized in Figure 1.

The study followed the Declaration of Helsinki, and ethical approval was obtained by the authors’ university’s ethics committee. Registration was made in the German National Register of Clinical Trials (DRKS00030616). Examinations were carried out in July 2023.

### Test instruments

Several tests were completed before and after the CGVR usage to evaluate the impact of the CGVR reaction tests on cognitive and motor performance and emotional status. These assessments included the Dementia Mood Picture Test^38^ to evaluate the IwD’s mood, the Trail-Making Test A (TMT-A)^39^ to assess the attention, a modified version of the State-Trait Anxiety Inventory^40^ to measure the state of anxiety, the Frailty and Injuries: Cooperative Studies of Intervention Techniques-4 (FICSIT-4)^41^ for balance performance, and the Timed-Up-and-Go Test (TUG)^42^ for mobility. While being in VR, a feedback questionnaire adapted from^24^ was completed by an instructor. After the CGVR tests, another feedback questionnaire was used to answer questions concerning the participants’ interest in CGVR and their just-made experience.

In the Dementia Mood Picture Test, participants choose a drawing that best represents their current mood from a set of faces depicting different emotions (negative mood, positive mood, cheerfulness, concern, sadness, anger).^38^ Deterioration in mood could be a sign of not accepting the VR.

During the TMT-A, the numbers 1-25 are presented and requested to be connected in ascending order as quickly as possible. The time needed and the number of mistakes are noted.^39^ Slower completion and more mistakes in the posttest could be associated with confusion induced by the CGVR.

The State-Trait Anxiety Inventory consists of several questions concerning the participants’ current and general anxiety levels. Previous applications of the inventory revealed that some questions (e.g., “I miss out on favorable opportunities because I can’t make up my mind quickly enough.’’) are hard to answer for IwD, which is why the inventory was shortened.^43^ The modified version encompassed 14 questions about the participants’ state anxiety, each rated with 1-4 points, which are summed up in the end. This results in a maximum of 56 points, where higher scores indicate a greater level of anxiety.^40^ Changes in anxiety after the CGVR would correspond to effects on the participants’ general well-being.

To perform the FICSIT-4, a sequence of four static balance tasks with increasing difficulty is completed with open and closed eyes. At first, the bilateral stance is requested, followed by the semi-tandem, tandem, and unilateral stance. Again, points are rewarded ranging between 0 points when the participant is not able to get in the stance starting position and four points when the stand is held unaided for 10 s. These points are summed up again, leading to a maximum total score of 28 ^41^. Fewer points in the post-test could be a sign of vertigo.

In the TUG, the participant sits on a chair and is tasked with standing up, walking three m, taking a 180° turn, walking the three m back, turning around and sitting down on the chair again. The time needed to complete this task is noted.^42^ The pre-and post-test comparison of the time needed for the TUG is made to assess the vertigo and dizziness of the participants.

While the participants were in VR, an instructor observed their behavior and administered a feedback questionnaire adapted from.^24^ The questions are related to the participants’ attention toward the VR, understanding of the given tasks, and the fun the participants express. Furthermore, it is noted whether the tasks were completed successfully. This questionnaire is used to evaluate the feasibility of the CGVR. Comprehensive information on the psychometric properties and methodological details of the test instruments can be found in the Additional File 1.

For the feedback questionnaire applied after the CGVR usage, 18 questions (like ‘Did you have fun during VR?’, for more, see Additional File 2) are rated by the participant between one and five points, where higher points represent a more positive answer. These questions are categorized into ‘Feedback to VR’ (questions 2, 3, 4, 5, 7, 9), ‘Interaction with VR’ (questions 1, 6, 8, 10, 11, 15, 16), and ‘Comfort’ (questions 12, 13, 14, 17, 18) Additional File 2. The points are summed up, leading to a maximum total score of 90 points. Additionally, open questions are asked like ‘What did you like most/least about the Virtual Reality experience?’. This questionnaire is applied to evaluate acceptance and negative side effects subjectively.

### Virtual reality setup

**Figure 2.**
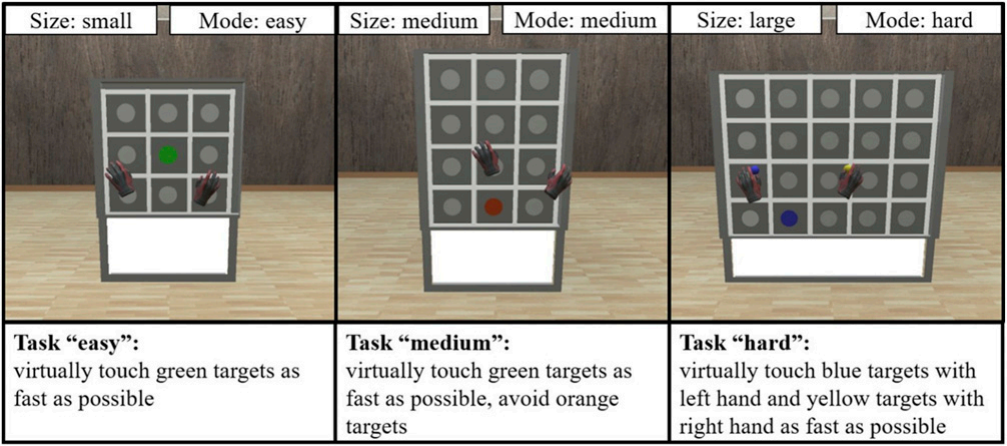
The three different modes and sizes of the reaction wall. All combinations of sizes and modes were possible, depending on the participant’s performance.

The virtual environment simulated a neutral gymnastic hall with a reaction wall and was modulated in Blender (version 3.2.1). Three different sizes of this reaction wall were developed, each with different amounts of targets (rows x columns): 3 x 3, 4 x 3, four x 5. The wall size was adjusted to these stimuli, which can be seen in Figure 2. The stimuli measured 10 cm in diameter, with a horizontal and vertical distance of 22.5 cm between their centers. The test’s behavior was developed with Unity (version 2021.3.11. f1), using self-written C#-scripts. SteamVR (version 1.26.5) transferred the virtual environment into the HMD (Pimax Vision 5k Super with 220° diagonal field of view, 2560 x 1440 pixels per eye, 90 Hz refreshing rate) and enabled intractability with the virtual components. The participants held two HTC VIVE Controllers (2018) to visualize the approximal position of their hands. These controllers and the HMD were tracked by two SteamVR Base Stations 2.0. The used laptop was equipped with an 11th-generation Intel Core i7 CPU, 16 GB DDR4 RAM, 1024 GB SSD, and an NVIDIA GeForce RTX 3060 graphic card (6 GB memory).

### Procedure

The following procedure was applied during the experiment.

**Figure 3.**
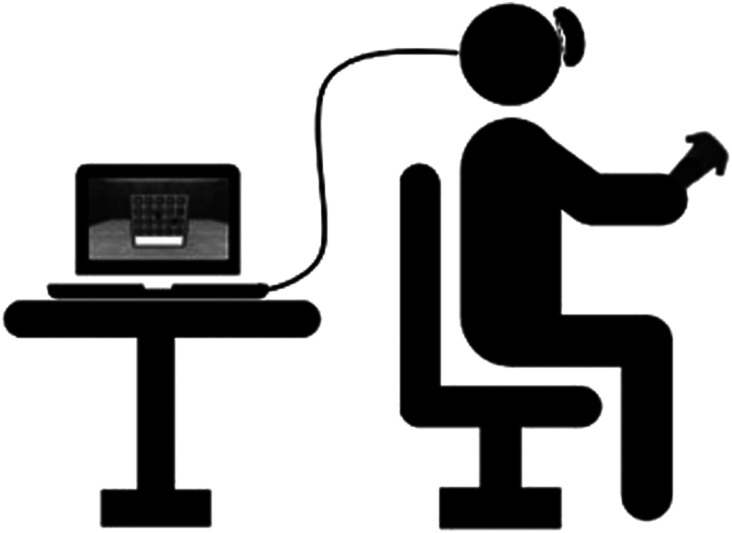
Participant sitting on a chair wearing the HMD. The HMD is connected to the computer via cable.

After the pre-test examinations, participants were seated in a chair and immersed in the virtual environment after being familiarized with the HMD (see Figure 3). The CGVR application consisted of reaction tests on a wall with three different sizes and modes, varying in difficulty, each lasted 45 s (Figure 2).

In the easy mode, several white stimuli are presented, from which one turns green and is supposed to be virtually touched with one of the controllers. Upon being touched, the stimulus turns white, and after a break of 0.4 s, one of the stimuli turns green again. The easy mode is completed three times, with the three different wall sizes, to identify the best fitting size concerning movement range and task complexity for each participant individually. This identified sized is used for the subsequent modes. In the medium mode, one white stimulus turns green (75 % chance) or orange (25 % chance). The green stimuli are supposed to be touched like in the easy mode, while orange stimuli must not be touched and turn white after 3 s. During the hard mode, one stimulus turns either blue or yellow. Blue stimuli are requested to be touched with the left hand, and yellow stimuli are requested to be touched with the right hand. As a memory aid, small spheres in the respective color are shown on the hands throughout the test. The modes and their outcome parameter are summarized in Table 1.

**Table 1.** Overview of the different modes and the corresponding outcome parameters.

Modus	Main task	Outcome parameter
Easy	Touching green stimuli	• Number of touched stimuli
		• Reaction time
		• Motor time
Medium	Touching green stimuli while avoiding orange stimuli	• Number of touched green stimuli
		• Number of mistakes (touched orange stimuli)
		• Reaction time
		• Motor time
Hard	Touching blue stimuli with the left hand and yellow stimuli with the right hand	• Number of correctly touched stimuli
		• Number of mistakes (touched stimuli with the wrong hand)
		• Reaction time
		• Motor time

Whenever a stimulus was touched, no matter if it was green, orange, blue or yellow, the touching controller vibrated shortly to provide haptic feedback.

The difficulty level of the tasks and the wall size were increased individually. The sizes are only increased if the participants can achieve all goals without any problems; larger sizes are skipped. If one mode was too difficult, the following modes were also skipped.

These adjustments were made possible by appropriate programming and modeling of the CGVR. This allowed changes to the mode or wall size to be made within seconds with just a few clicks, further highlighting the advantages of the CGVR. The procedure concerning size and difficulty increasement is visualized in Figure 1.

After completing the last mode, the HMD was removed from the participant’s head, and the feedback questionnaire was administered. Subsequently, the post-test measurements started.

During the testing, two test administrators were always present. This served, on the one hand, to ensure the safety of this particularly vulnerable group of participants, and on the other hand, to facilitate the implementation of the intervention. One person operated the VR system, was able to see in real time what the participant was experiencing, and could immediately adjust the intervention if technical problems or errors occurred. This also represents an additional advantage of computer-generated VR.

### Virtual reality data collection

Several parameters are computed during the reaction test in CGVR (see Table 1) which are calculated from the following data.

For all three modes, the time point when a correct stimulus was touched, the hand used, the time point when the stimulus was rendered in the HMD, the stimulus column on the reaction wall, and the total number of touched stimuli within 45 s are saved. Additionally, for the medium and hard modes, the stimulus color and number of mistakes are recorded.

Furthermore, during VR usage, the position of the stimulus that is supposed to be touched and the position and rotation of both controllers and the HMD are constantly recorded at 90 Hz. These data are used to calculate the reaction and motor times toward each stimulus. Reaction time is the time between the stimulus being rendered in the HMD and the first controller movement toward this stimulus. In contrast, motor time is the time between the first controller movement toward the stimulus and the stimulus being touched.

Additionally, the fulfillment of the different modes is translated into a modus score. Independent completion of a mode warrants one point, while half a point is granted if assistance is required. Overall, a maximum of five points is attainable through autonomous completion of the easy mode across three wall sizes along with the medium and hard modes.

### Statistical analysis

Statistical data analysis was performed using SPSS, version 28 (IBM). The pre-post-examinations (State-Trait Anxiety Inventory, TMT-A, FICSIT-4, and TUG) were analyzed using paired t-tests. The significance level was set to α = 0.05. To avoid alpha error computation, the significance level was adjusted using the Bonferroni-Holm correction. Cohen’s classification was used to interpret the effect sizes (d = 0.2/r = 0.1, small; d = 0.5/r = 0.3, moderate; d = 0.8/r = 0.5, large). The n-numbers in the results differ because not all participants were able to complete all the tasks.

In addition, correlations were calculated to assess the feasibility of the individual modes. The Pearson correlation coefficient (r) was calculated between MMSE and mode score, reaction time and motor time.

The Dementia Mood Picture Test, MMSE and feedback questionnaire were analyzed descriptively.

## Results

### Sample characteristics

Three subjects dropped out during the study because of disinterest (n = (2) and disturbance by the HMD (n = 1), corresponding to a drop-out rate of 9 %. Thus, 29 out of 32 IwD were analyzed.

The sample characteristics are presented in Table 2. The HMD could also be used with visual and hearing aids.

**Table 2.** Sample characteristics.

Variables	N = 29
	M±SD/n (%)
Demographics	
Age (years)	83.41±5.09
Gender n (%)	f = 23 (79.3)
	m = 6 (20.7)
Health assessments	
Nausea n (%)	Yes = 2 (6.9)
	No = 27 (93.1)
Dizziness n (%)	Yes = 5 (17.2)
	No = 24 (82.8)
Visual impairments n (%)	Yes = 22 (75.9)
	No = 7 (24.1)
Hearing impairments n (%)	Yes = 11 (37.9)
	No = 18 (62.1)
VR experience before study	
Heard of VR n (%)	Yes = 4 (13.8)
	No = 25 (86.2)
Used VR before n (%)	Yes = 2 (6.9)
	No = 27 (93.1)
Cognitive assessments	
MMSE score	19.07±5.32
Stage of dementia (mild) n (%)	16 (55.2)
Stage of dementia (moderate) n (%)	12 (41.4)
Stage of dementia (severe) n (%)	1 (3.4)
Physical assessments	
Mobility aids usage n (%)	Yes = 14 (48.3)
	No = 15 (51.7)

Notes. M: mean values; SD: standard deviations; m: male; f: female; MMSE: Mini-Mental State Examination; VR: Virtual Reality.

### Outcome parameters

#### Feasibility

The observational data gathered in the feedback questionnaire while utilizing CGVR indicated that approximately 80 % of the participants demonstrated attentiveness and engaged actively with the CGVR. Furthermore, about 80 % of the subjects displayed a high or moderate level of comprehension regarding the tasks associated with specific modes, and were able to execute these tasks effectively. The observational records further revealed that 90 % of the participants exhibited no signs of boredom and experienced considerable enjoyment.

**Figure 4.**
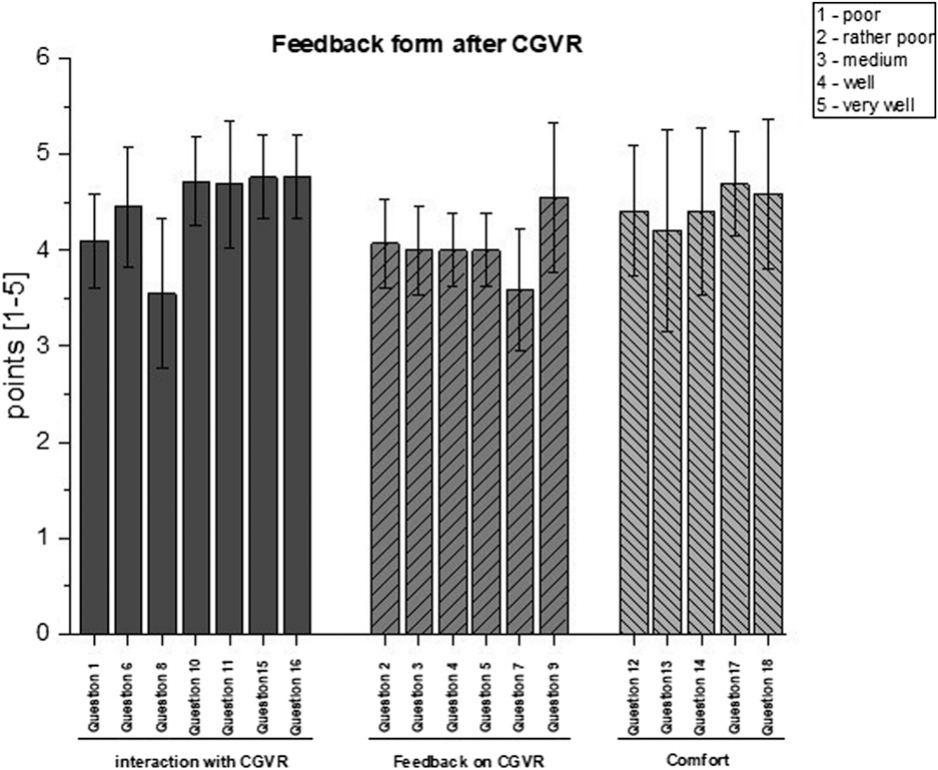
Results of the feedback form applied after the CGVR exposure, divided into the individual dimensions. Questions can be found in the Additional File 1.

The feedback form following^24^ additionally showed that on average the subjects rated the CGVR as ‘good’ with a total score of 77.5 ± 6,7 from 90 possible points: interaction (31,4/35 points), feedback (24,2/30 points), and comfort (22,3/25 points) (see Figure 4). The questions in the three dimensions can be found in the Additional File 2. The mood of the participants was not altered by the CGVR experience since similar results were recorded in the Dementia Mood Picture test after the reaction tests in CGVR (all participants exhibited a positive mood).

In addition, the actual time spent in CGVR was recorded. It was shown that a mean of 496 s (8.30 min) was spent in the CGVR (maximum was 540 s (9 min)).

The feasibility of the distinct modes is presented in Table 3. This table illustrates that the easy mode with the small wall was completed by all participants independently or with assistance. However, as the test advanced and the level of difficulty increased due to the wall’s size or the task’s complexity, the number of participants completing the tasks without any issues diminished. In mode 3, which was designed as the most challenging mode, only 72.4 % of participants could finish the task autonomously or with support.

**Table 3.** Feasibility of the modes.

Task description (n = 29)	Mode 1	Mode 1	Mode 1	Mode 2	Mode 3
	3x3 T-Wall	4x3 T-Wall	4x5 T-Wall		
Feasible (n, %)	25 (86.2 %)	24 (82.8 %)	24 (82.8 %)	19 (65.5 %)	19 (65.5 %)
Partial (n, %)	4 (13.8 %)	4 (13.8 %)	4 (13.8 %)	4 (13.8 %)	2 (6.9 %)
Not feasible (n, %)	0 (0.0 %)	1 (3.4 %)	1 (3.4 %)	6 (20.7 %)	8 (27.6 %)

Notes. Partial Indicates completion with assistance such as verbal prompts or acoustic support.

Several correlations were established to elucidate the feasibility of the different modes. The analysis revealed a strong correlation between the MMSE score and the mode score (4.14 ± 1.38) (r = 0.773; p < .001). Furthermore, a moderate correlation (r = −0.425, p = .024) between the MMSE score and reaction time and a strong correlation (r = −0.560, p = .002) between the MMSE score and motor reaction time was observed.

#### Impact on motor, cognitive, and mental state

To rule out potential adverse effects, participants’ motor and cognitive functions, as well as their mental state, were evaluated before and after the VR intervention. The results show no significant changes in cognitive and motor performance and mental state after CGVR (Table 4). However, almost all parameters revealed slight but not statistically significant improvements after CGVR. On the TMT-A, IwD improved by a mean of 5.76 s, the state anxiety score by 0.41 points, and the FICSIT-4 score by 0.29 points from pre-to post (Table 4). The results of the TUG slightly decreased by 0.21 s.

**Table 4.** Results of pre-and post-test.

Outcomes	Pre-test	Post-test	t-Test	p (Bonferroni-holm)	Effect size (d)
	M ± SD (Cl_95_)	M ± SD (Cl_95_)	z-value		
TMT-A (s) n = 22	86.05 ± 30.8 (72.38 – 99.72)	80.29 ± 31.7 (66.23 – 94.34)	1.349	.096 (.29)	.288
State anxiety (points) n = 29	24.48 ± 4.6 (22.73 – 26.24)	24.07 ± 4.3 (22.45 – 25.69)	1.440	.080 (.32)	.267
FICSIT-4 (points) n = 24	14.38 ± 3.9 (12.75 – 16.00)	14.67 ± 3.9 (13.02 – 16.32)	−1.071	.148 (.30)	.219
TUG (s) n = 25	15.34 ± 6.2 (12.77 – 17.92)	15.13 ± 5.6 (12.81 – 17.45)	−0.396	.348 (.348)	.079

Note. M: Mean value; SD: standard deviations; Cl_95_: 95% confidence interval; TMT-A: Trail-Making Test A; FICSIT-4: Frailty and Injuries: Cooperative Studies of Intervention Techniques; TUG: Timed-Up-and-Go Test.

## Discussion

The integration of digital solutions, including computer-generated virtual reality (CGVR) environments, into dementia care opens new possibilities for personalized therapeutic approaches. Existing studies already show positive outcomes associated with the use of VR. The aim of this feasibility study was to examine the applicability of a CGVR application for IwD, focusing on acceptance, safety, immediate effects on well-being, and potential negative effects on motor and cognitive functions. This study served as a preliminary investigation for a potential subsequent randomized controlled trial (RCT). The CGVR application was generally well received, as evidenced by high feedback scores, which reflect a positive evaluation of the CGVR experience. No participant reported dizziness, nausea, confusion, or disorientation, indicating no adverse effects on well-being. These findings are consistent with results from studies using immersive VR, where participants did not experience nausea or other side effects such as dizziness, disorientation, or confusion during use.^22,28,44^ Overall, the response to CGVR use can therefore be assessed as similarly positive to that of previously studied immersive VR environments. Positive resonance was also evident in participant feedback such as “The virtual world seemed very real,” “It was fascinating to observe,” or “I would like to spend more time in this environment.”

Questions concerning wearing comfort primarily related to the use of the HMD. In most cases, the five questions were answered in a way that indicated no significant discomfort from the device (22.3 out of a possible 25 points). Prior experience with HMDs cannot explain the overall high comfort scores, as only 6.9% of participants had previously worn such a device.

The low dropout rate in this study, comparable to,^24^ suggests that CGVR was well accepted and easily implemented by participants. The average duration of use further supports its feasibility. Additionally, almost all participants were able to complete the “simple modes” with three different wall sizes independently or with minimal assistance.

Unlike,^24^ whose study employed 360° videos, the present study demonstrated that CGVR can be used without difficulty in IwD. Notably, unlike 360° videos—which, although offering higher realism, severely limit interactivity^27^—CGVR has rarely been discussed so far.^25,27,31^ A key advantage of computer-generated environments lies in their capacity for customization. This adaptability is particularly important for IwD, who benefit significantly from personalized interventions. Static 360-degree videos provide this flexibility only to a limited extent. Through the implementation of different modes in our reaction wall, we were able to represent a range of difficulty levels. Our correlation analysis demonstrated that this customization is indeed feasible and meaningful. The results showed that feasibility varied depending on the difficulty level of the CGVR modes: as difficulty increased, success rates decreased. Significant correlations were observed between MMSE scores and successful completion of the modes, as well as reaction and motor response times, indicating that cognitive status influences performance in the CGVR task. This highlights the importance of individualized approaches in digital health interventions for IwD.^16^ CGVR offers clear advantages in this context due to its high flexibility and adaptability. As our study has shown, the size of virtual objects can be adjusted to individual abilities within seconds, requiring more or less movement. In addition, visual stimuli can be easily modified in terms of size, speed, or contrast to suit the abilities and current physical or cognitive state of the participants.

CGVR may offer added value in this context and potentially exert a positive influence on cognitive and motor functions. This supports the hypothesis that CGVR environments could serve as a suitable basis for customizable training interventions.^21^

No significant changes in cognitive or motor performance or psychological state were observed following the CGVR session, although slight, statistically non-significant improvements were noted across nearly all parameters. Since the primary aim of the study was to assess potential adverse effects, the absence of significant changes can already be considered a positive finding. Furthermore, the lack of significant improvements was unsurprising, as the study involved only a short, single-session exposure, which was not intended to produce lasting effects. However, the slight non-significant improvements align with the findings of,^26^ who concluded that VR may be a promising non-pharmacological method to enhance cognitive and motor skills in IwD. Moreover, these trends may indicate potential positive effects with extended use—an aspect that should be addressed in future studies. Particularly noteworthy regarding motor outcomes is the fact that the CGVR was conducted entirely in a seated position, without standing or walking exercises. Future CGVR interventions incorporating more physical activity could potentially yield broader effects.

Concerns are often raised that CGVR may induce confusion in IwD who are prone to disoriented states,^18^ which can be associated with extremely negative emotions.^45^ However, in the present study, no participant showed signs of fear, confusion, nausea, or dizziness, supporting the findings of^28^ (CGVR with low display resolution) regarding the absence of negative sensations during VR use. These observations are crucial for the long-term application of CGVR, as sustainable implementation in care facilities is only conceivable if the technology provides added value alongside emotional tolerability. The observed joy and attention, in combination with the absence of fear, confusion, nausea, or dizziness, also suggest a potential impact on quality of life. CGVR could also provide support for managing challenging behaviors in home-based dementia care.^46^

The instruments used to assess mood (Dementia Mood Picture Test, DMPT) and state anxiety (State-Trait Anxiety Inventory, STAI) are well-established and have been successfully applied in older adults, including some with dementia.^43,47^ Nevertheless, there are limitations to interpretation, as both instruments rely on self-report, which may be affected by cognitive impairments—such as reduced self-awareness or expressive ability. The DMPT was specifically developed for individuals with severe cognitive impairment and is considered practical in application, though its sensitivity to short-term affective changes has not yet been fully established.^47^ The STAI may also face validity limitations in advanced dementia.^43^ Future studies could complement these tools with observational measures, such as the Dementia Mood Assessment Scale (DMAS) or the Observed Emotion Rating Scale (OERS), as well as physiological parameters (e.g., heart rate, skin conductance), to more comprehensively and objectively capture emotional responses.

The results of this feasibility study underscore the potential of CGVR as a non-pharmacological intervention for IwD and reflect the growing interest in digital health solutions in dementia care.^16^ The positive reception and engagement of participants suggest that CGVR could represent a feasible and enjoyable activity for individuals with mild to moderate dementia.^21,24^ Our findings indicate that such interventions do not trigger adverse side effects and therefore offer a promising foundation for subsequent randomized controlled trials.

Despite these promising findings, the study also highlights the need for further research to evaluate the long-term effects and efficacy of CGVR interventions in dementia care.^4^ points out that non-pharmacological measures are increasingly recognized for their value in dementia therapy. However, specific interventions—including CGVR—require a broader evidence base through larger, long-term studies. Furthermore, future studies should address cognitive and motor training for people with disabilities conducted in CGVR. This should exploit the full potential of CGVR, particularly its ability to quickly adapt to individual abilities such as range of motion and cognitive status.

## Limitations

As with any study, certain limitations should be considered. First, the CGVR application did not encourage or elicit physical activity. This decision was made to initially investigate the general feasibility and gather experience with CGVR in IwD without overloading the participants and ensuring safe exposure to CGVR. Additionally, the small sample size, while being appropriate given the study design and objectives, limits the generalizability of the findings.

The single-use of CGVR in a seated position and the relatively mild degree of dementia in the sample are further limitations. These constraints were necessary to ensure safe first-time use but may applicability of the results to more varied or advanced dementia cases.

Moreover, the study design involved only one session of CGVR exposure, which may not be sufficient to fully exclude negative side effects. This limitation suggests that long-term studies with repeated sessions are necessary to assess the full safety and, additionally, potential positive effects of CGVR in dementia care.

## Conclusion

In summary, this feasibility study provides valuable insights into the applicability of CGVR as a therapeutic tool for IwD. It represents a first empirical contribution to the structured development of movement-based CGVR interventions in dementia care. The results demonstrate high acceptance and indicate potentially positive effects on subjective well-being. These findings expand the understanding of VR, particularly in the context of CGVR, and support the potential of CGVR – and VR more generally – as a complementary, non-pharmacological intervention in dementia care.^16,21,44^ Notably, the absence of adverse effects on cognitive and motor functions highlights the importance of further research into the long-term effectiveness and targeted implementation of CGVR to inform evidence-based practice. While initial results regarding CGVR applications in dementia therapy are promising, the field remains in its early stages. This study addresses a key research deficit, as identified by^22^ and,^21^ and provides initial data on the feasibility and safety of movement-based CGVR applications in IwD. The present study corroborates and extends previous research by employing a larger sample size and incorporating more complex motor tasks than those used in most prior studies.^30–34^ The study focused exclusively on reaction tasks within a CGVR environment. Future research should therefore also explore applications involving daily living activities and systematically investigate movement-based CGVR use in IwD. The findings of this feasibility study underscore the complexity of evaluating technological interventions in this population, particularly in light of the diverse needs and abilities of people with dementia.^22,23,44^ The positive feedback, high participation rate, and absence of negative side effects support the need for further studies examining the long-term use of CGVR – for example, to enhance cognitive and motor functions through interactive elements such as the reaction wall developed in this study. These findings offer a foundation for the integration of CGVR into structured care settings and underline its potential as a meaningful, individualized, and engaging component of dementia therapy.

## Supplemental Material

Supplemental Material - Assessing the feasibility of interactions within a computer-generated virtual reality for people with dementia Supplemental Material for Assessing the feasibility of interactions within a computer-generated virtual reality for people with dementia by Alexander Prinz, Dan Bürger, Marvin Schmidt, Richard Jesse, Kerstin Witte in Journal of Rehabilitation and Assistive Technologies Engineering.

Supplemental Material - Assessing the feasibility of interactions within a computer-generated virtual reality for people with dementia Supplemental Material for Assessing the feasibility of interactions within a computer-generated virtual reality for people with dementia by Alexander Prinz, Dan Bürger, Marvin Schmidt, Richard Jesse, Kerstin Witte in Journal of Rehabilitation and Assistive Technologies Engineering.

## Data Availability

The datasets used and/or analyzed during the current study are available from the corresponding author on reasonable request.*

## Abbreviations

IwD: individuals living with dementia
VR: Virtual Reality
CGVR: computer generated Virtual Reality
HMD: Head-mounted Display
TMT-A: Trail-Making Test A
FICSIT-4– Frailty and Injuries: Cooperative Studies of Intervention Techniques-4
TUG: Timed-Up-and-Go Test

## References

[1] GBD 2019 Dementia Forecasting Collaborators . Estimation of the global prevalence of dementia in 2019 and forecasted prevalence in 2050: an analysis for the global burden of disease study 2019. Lancet Public Health 2022; 7: e105–e125. 34998485 10.1016/S2468-2667(21)00249-8 PMC8810394

[2] World Health Organization . Global status report on the public health response to dementia. World Health Organization, 2021. https://apps.who.int/iris/rest/bitstreams/1367115/retrieve (accessed 8 April 2024).

[3] Cummings JL Morstorf T Zhong K . Alzheimer's disease drug-development pipeline: few candidates, frequent failures. Alzheimers Res Ther 2014; 6: 37. 25024750 10.1186/alzrt269 PMC4095696

[4] Tesky VA Schall A Pantel J . Nichtmedikamentöse Interventionen für Menschen mit Demenz. Inn Med 2023; 64: 139–146. 10.1007/s00108-022-01446-1 36520205

[5] López-Ortiz S Lista S Valenzuela PL , et al. Effects of physical activity and exercise interventions on Alzheimer's disease: an umbrella review of existing meta-analyses. J Neurol 2023; 270: 711–725. 36342524 10.1007/s00415-022-11454-8

[6] Mendes A Bergh S Cesana BM , et al. Respectful caring for the agitated elderly (ReCAGE): a multicentre, prospective, observational Study to evaluate the effectiveness of special care units for people with dementia. J Alzheimers Dis 2023; 96: 1083–1096. 37927262 10.3233/JAD-230708

[7] Prinz A Schumacher A Witte K . Changes in selected cognitive and motor skills as well as the quality of life after a 24-Week multidimensional music-based exercise program in people with dementia. Am J Alzheimers Dis Other Demen 2023; 38: 15333175231191022. 37611012 10.1177/15333175231191022 PMC10655793

[8] Teri L Gibbons LE McCurry SM , et al. Exercise plus behavioral management in patients with alzheimer disease: a randomized controlled trial. JAMA 2003; 290: 2015–2022. 14559955 10.1001/jama.290.15.2015

[9] Logsdon RG McCurry SM Teri L . Evidence-based interventions to improve quality of life for individuals with dementia. Alzheimers Care Today 2007; 8: 309–318. 19030120 PMC2585781

[10] Liang Y-J Su Q-W Sheng Z-R , et al. Effectiveness of physical activity interventions on cognition, neuropsychiatric symptoms, and quality of life of alzheimer's disease: an update of a systematic review and meta-analysis. Front Aging Neurosci 2022; 14: 830824. 35309887 10.3389/fnagi.2022.830824 PMC8926300

[11] Liao Y-Y Chen I-H Lin Y-J , et al. Effects of virtual reality-based physical and cognitive training on executive function and dual-task gait performance in older adults with mild cognitive impairment: a randomized control trial. Front Aging Neurosci 2019; 11: 162. 31379553 10.3389/fnagi.2019.00162 PMC6646677

[12] Zhou S Chen S Liu X , et al. Physical activity improves cognition and activities of daily living in adults with alzheimer's disease: a systematic review and meta-analysis of randomized controlled trials. Int J Environ Res Publ Health 2022; 19: 1216. 10.3390/ijerph19031216 PMC8834999 35162238

[13] van Alphen HJM Hortobágyi T van Heuvelen MJG . Barriers, motivators, and facilitators of physical activity in dementia patients: a systematic review. Arch Gerontol Geriatr 2016; 66: 109–118. 27295140 10.1016/j.archger.2016.05.008

[14] Diener J Rayling S Bezold J , et al. Effectiveness and acceptability of e- and m-Health interventions to promote physical activity and prevent Falls in nursing Homes-A systematic review. Front Physiol 2022; 13: 894397. 35669573 10.3389/fphys.2022.894397 PMC9163679

[15] Sohn M Yang J Sohn J , et al. Digital healthcare for dementia and cognitive impairment: a scoping review. Int J Nurs Stud 2023; 140: 104413. 36821951 10.1016/j.ijnurstu.2022.104413

[16] Biskupiak Z Ha VV Rohaj A , et al. Digital therapeutics for improving effectiveness of pharmaceutical drugs and biological products: preclinical and clinical studies supporting development of drug + digital combination therapies for chronic diseases. J Clin Med 2024; 13: 403. 38256537 10.3390/jcm13020403 PMC10816409

[17] Rose V Stewart I Jenkins KG , et al. Bringing the outside in: the feasibility of virtual reality with people with dementia in an inpatient psychiatric care setting. Dementia 2021; 20: 106–129. 31510801 10.1177/1471301219868036

[18] Seifert A Schlomann A . The use of virtual and augmented reality by older adults: potentials and challenges. Front Virtual Real 2021; 2: 639718.

[19] Pantelidis VS . Reasons to use virtual reality in education and training courses and a model to determine when to use virtual reality. Themes in Science and Technology Education 2009; 2: 59–70.

[20] Dargar S Kennedy R Lai W , et al. Towards immersive virtual reality (iVR): a route to surgical expertise. J Comput Surg 2015; 2: 2. 26478852 10.1186/s40244-015-0015-8 PMC4606894

[21] Wiebe A Kannen K Selaskowski B , et al. Virtual reality in the diagnostic and therapy for mental disorders: a systematic review. Clin Psychol Rev 2022; 98: 102213. 36356351 10.1016/j.cpr.2022.102213

[22] Strong J . Immersive virtual reality and persons with dementia: a literature review. J Gerontol Soc Work 2020; 63: 209–226. 32091323 10.1080/01634372.2020.1733726

[23] Prinz A Bürger D Krafft J , et al. The use of immersive virtual reality in nursing homes for people with dementia under consideration of cognitive and motor performance as well as emotional reaction: a feasibility study. JMIR XR Spatial Comput. 2024; 1: e54724. doi:10.2196/54724. PMC13179109 42147211

[24] Appel L Appel E Bogler O , et al. Older adults with cognitive And/or physical impairments can benefit from immersive virtual reality experiences: a feasibility Study. Front Med 2019; 6: 329. 10.3389/fmed.2019.00329 PMC6974513 32010701

[25] Rodríguez-Mansilla J Chamizo-Gallego P González-Sánchez B , et al. Virtual reality as a complementary therapy in the rehabilitation of balance and gait disorders in patients with mild cognitive impairment and alzheimer's disease: systematic review. Clin Rehabil 2025; 39: 728–739. 40266568 10.1177/02692155251328619

[26] Zhu S Sui Y Shen Y , et al. Effects of virtual reality intervention on cognition and motor function in older adults with mild cognitive impairment or dementia: a systematic review and meta-analysis. Front Aging Neurosci 2021; 13: 586999. 34025384 10.3389/fnagi.2021.586999 PMC8136286

[27] Brivio E Serino S Negro Cousa E , et al. Virtual reality and 360° panorama technology: a media comparison to study changes in sense of presence, anxiety, and positive emotions. Virtual Real 2021; 25: 303–311.

[28] Kim J-H Park S Lim H . Developing a virtual reality for people with dementia in nursing homes based on their psychological needs: a feasibility study. BMC Geriatr 2021; 21: 167. 33678160 10.1186/s12877-021-02125-w PMC7938563

[29] Huang L-C Yang Y-H . The long-term effects of immersive virtual reality reminiscence in people with dementia: longitudinal observational study. JMIR Serious Games 2022; 10: e36720. 35877169 10.2196/36720 PMC9361147

[30] Eisapour M Cao S Boger J . Game design for users with constraint. In: Adjunct Proceedings of the 31st Annual ACM Symposium on User Interface Software and Technology. (ed Baudisch P Schmidt A Wilson A ), ACM. 2018, pp. 128–130.

[31] Flynn A Barry M Qi Koh W , et al. Introducing and familiarising older adults living with dementia and their caregivers to virtual reality. Int J Environ Res Publ Health 2022; 19: 16343. 10.3390/ijerph192316343 PMC9736737 36498417

[32] Flynn A Koh WQ Reilly G , et al. A multi-user virtual reality social connecting space for people living with dementia and their support persons: a participatory action research study. Int J Hum Comput Interact 2024: 1–19.

[33] Kruse L Karaosmanoglu S Rings S , et al. A long-term user study of an immersive exergame for older adults with mild dementia during the COVID-19 pandemic, 2021.

[34] Muñoz J Mehrabi S Li Y , et al. Immersive virtual reality exergames for persons living with dementia: user-centered design study as a multistakeholder team during the COVID-19 pandemic. JMIR Serious Games 2022; 10: e29987. 35044320 10.2196/29987 PMC8772876

[35] Crimin K Allen PJ Abba I , et al. Identifying predictive factors of patient dropout in Alzheimer’s disease clinical trials. Alzheimer's Dement 2021; 17: e052361.

[36] Ritchie M Gillen DL Grill JD . Estimating attrition in mild-to-moderate alzheimer's disease and mild cognitive impairment clinical trials. Alzheimers Res Ther 2023; 15: 203. 37990339 10.1186/s13195-023-01352-0 PMC10662394

[37] Folstein MF Folstein SE McHugh PR . Mini-mental state A practical method for grading the cognitive state of patients for the clinician. J Psychiatr Res 1975; 12: 189–198. 1202204 10.1016/0022-3956(75)90026-6

[38] Tappen RM Barry C . Assessment of affect in advanced alzheimer's disease: the dementia mood picture test. J Gerontol Nurs 1995; 21: 44–46. 7706649 10.3928/0098-9134-19950301-09

[39] Reitan RM Reitan RM Trail making test: manual for administration and scoring. 1992.

[40] Spielberger CD Gorsuch RL Lushene RE . Manual for the State--‐Trait STAI manual for the state-trait anxiety inventory. Consulting Psychologists Press, 1970.

[41] Rossiter-Fornoff JE Wolf SL Wolfson LI , et al. A cross-sectional validation study of the FICSIT common data base static balance measures. Frailty and injuries: cooperative studies of intervention techniques. J Gerontol A Biol Sci Med Sci 1995; 50: M291–M297. 7583799 10.1093/gerona/50a.6.m291

[42] Podsiadlo D Richardson S . The timed “Up & Go”: a test of basic functional mobility for frail elderly persons. J Am Geriatr Soc 1991; 39: 142–148. 1991946 10.1111/j.1532-5415.1991.tb01616.x

[43] Fernández-Blázquez MA Ávila-Villanueva M López-Pina JA , et al. Psychometric properties of a new short version of the state-trait anxiety inventory (STAI) for the assessment of anxiety in the elderly. Neurologia 2015; 30: 352–358. 24484757 10.1016/j.nrl.2013.12.015

[44] Appel L Ali S Narag T , et al. Virtual reality to promote wellbeing in persons with dementia: a scoping review. J Rehabil Assist Technol Eng 2021; 8: 20556683211053952. 35024166 10.1177/20556683211053952 PMC8743938

[45] Kar N . Behavioral and psychological symptoms of dementia and their management. Indian J Psychiatry 2009; 51 Suppl 1(Suppl 1): S77–S86. 21416023 PMC3038531

[46] Feast A Orrell M Charlesworth G , et al. Behavioural and psychological symptoms in dementia and the challenges for family carers: systematic review. Br J Psychiatry 2016; 208: 429–434. 26989095 10.1192/bjp.bp.114.153684 PMC4853642

[47] Clarke C Woods B Moniz-Cook E , et al. Measuring the well-being of people with dementia: a conceptual scoping review. Health Qual Life Outcome 2020; 18: 249. 10.1186/s12955-020-01440-x PMC7382062 32709238

