# Supplementary material for: Assessing the feasibility of interactions within a computer-generated virtual reality for people with dementia
Source: J Rehabil Assist Technol Eng. 2025 Nov 27;12:20556683251393992. doi: 10.1177/20556683251393992 (PMC12660644; doi:10.1177/20556683251393992)
Supplement: Supplemental Material - Assessing the feasibility of interactions within a computer-generated virtual reality for people with dementia [file sj-pdf-1-jrt-10.1177_20556683251393992.pdf]

**Table 1** cognitive test procedures

<table>
<tr><th colspan="2">cognition</th></tr>
<tr><th colspan="2">Mini-Mental State Examination ( Folstein, Folstein & McHugh, 1975)</th></tr>
<tr><th>Cognitive area</th><th>Declaration</th></tr>
<tr><td>General cognitive function level (Screening)</td><td>- Answering questions to determine the cognitive abilities of older people<br>o Orientation<br>o Absorption capacity<br>o Attention and calculation<br>o Memory<br>o Language<br>o constructive practice<br>- - maximum 30 points</td></tr>
<tr><td colspan="2">Quality criteria:<br><br>- Reliability: Interrater reliability: ICC = 0.83 - 0.95<br>- Test-restest reliability: $r_{tt}$=0.84<br>-<br>- Validity:<br>o Criterion validity: Relatively good differentiation between healthy and demented individuals, but less sensitive in the MCI domain.<br>o Construct validity: moderate correlation with other screening and rating methods (r=0.43 - 0.70 with DemTect, r= 0.75 - 0.78 with SKT, r= 0.55 - 0.76 with ADAS-Cog) and Everyday competence (r= 0.28 with B-ADL)</td></tr>
<tr><th colspan="2">Trail-Making- Test A</th></tr>
<tr><th>Cognitive area</th><th>Declaration</th></tr>
<tr><td>Psychomotor speed,<br><br>Executive functions</td><td>- number linking as fast as possible (from 1-25)<br>- - max. 180 s</td></tr>
<tr><td colspan="2">Quality criteria:<br>- Test-restest reliability: $r_{tt}$= 0.76 - 0.89 (Wagner et al., 2011)<br>- interrater reliability of the TMT-A is implicitly very high, as it is an objective, time-based test with clear scoring guidelines<br><br>Validity:<br>- The Construct validity of the TMT-A is well documented by significant correlations with established neuropsychological tests (Digit Symbol Subtest, finger tapping test, Digit Backward, Stroop Color-Word scores) (Sanchez-Cubillo et al., 2009)<br>- Criterion validity: TMT-A can effectively differentiate between people with and without organic brain damage (Reitan, 1958)</td></tr>
</table>

| **State-Trait Anxiety Inventory ( Laux, Glanzmann, Schaffner & Spielberger, 1981)** | |
|---|---|
| **Area** | **Declaration** |
| Fear as a state and fear as a property | - two scales of the STAI with 20 items each are used to capture anxiety as a state (state anxiety) and anxiety as a trait (trait anxiety)<br>- 4s Likert scale<br>- Sum values from 20 - 80, where higher values stronger expressions of the respective anxiety trait show |
| Quality criteria:<br><br>- Internal consistency for both scales α = 0.90<br>- Test-restest reliability Trait anxiety: r=0.77 - 0.90<br>- Test-restest reliability State anxiety: r= 0.22 - 0.53<br>-<br>- Validity:<br>    o Criterion validity: determined by correlation with other test scales (including FPI, EPI, EWL, BIV, Paranoid Depression Scale, Complaints List, and various subjective stress scales)<br>    o Correlations of the Trait Anxiety Scale with the Lück and Timaeus Manifest Anxiety Measurement Scale (MAS) ranged from r = .73 to r = .90 | |

| **Dementia Mood Picture Test (Tappen & Barry, 1995)** | |
|---|---|
| **Area** | **Declaration** |
| Assessment of mood and affective state in individuals with severe cognitive impairment | - Visual recognition of six basic emotions (e.g., happiness, sadness, anger) using pictorial cards<br>- Self-assessment supported by visual cues<br>- Designed for persons with advanced dementia and limited verbal communication<br>- Selection of emotion pictures corresponding to current mood; results qualitatively interpreted |
| Quality criteria:<br><br>- Interrater reliability: ICC = 0.95 – 1.00 (Tappen & Barry, 1995)<br>- Validity:<br>    o Criterion validity:<br>    o Correlation with Montgomery-Åsberg Depression Rating Scale: r = –0.51 (Tappen & Barry, 1995) | |

**Table 2** motor skills test procedure

| **Motor function** | | |
|---|---|---|
| **Test procedure** | **Motor area** | **declaration** |
| Timed-Up-and-Go-Test<br><br>(D. Podsiadlo & S. Richardson, 1991) | Mobility restriction | The test person is to stand up from a sitting position without assistance, walk back and forth for 3 meters and sit down again (aids are allowed)<br><br>Score from ≤10s (Everyday mobility unrestricted) to ≥30 (Pronounced mobility restriction, usually intervention/ Need for assistive devices) |
| | Quality criteria:<br>• Reliability: Interrater reliability: ICC = 0.94 – 0.99 (Kristensen et al., 2011)<br>  o Test-retest reliability: ICC = 0.96 - 0.99 (Flansbierr,2005)<br>• Validity:<br>  o Criterion validity: Berg Balance Scale (r=0.81), Barthel Index (r=0.78) (Podsiadlo, 1991) | |
| FICSIT-4<br><br>(Rossiter-Fornoff et al. 1995) | Static balance | The subject must perform four different stances with eyes open and closed (parallel, semi-tandem, tandem, and one-legged stance tests).<br><br>Each stance is performed for a maximum of 10 seconds and then scored on a 5 point scale (0 points need help to keep from falling to 4 points able to stand 10 seconds safely). At the end, all the points obtained from the stands are added up to a total score. |
| | Quality criteria:<br>• Reliability: Test-rests-reliability was good (r=.66) (Rossiter-Fornoff et al., 1995)<br>• Validity:<br>  o Content validity was moderate (r=.20 - .52) (Rossiter-Fornoff et al., 1995) | |

## Reference

Sánchez-Cubillo, I., Periáñez, J. A., Adrover-Roig, D., Rodríguez-Sánchez, J. M., Ríos-Lago, M., Tirapu, J., & Barceló, F. (2009). Construct validity of the Trail Making Test: Role of task-switching, working memory, inhibition/interference control, and visuomotor abilities. *Journal of the International Neuropsychological Society, 15*(3), 438–450. https://doi.org/10.1017/S1355617709090626

Wagner, S., Helmreich, I., Dahmen, N., Lieb, K., & Tadic, A. (2011). Reliability of three alternate forms of the Trail Making Tests A and B. *Archives of Clinical Neuropsychology, 26*(4), 314–321. https://doi.org/ 10.1093/arclin/acr024

Reitan, R. M. (1958). Validity of the Trail Making Test as an indicator of organic brain damage. *Perceptual and Motor Skills, 8*(3), 271–276. https://doi.org/10.2466/pms.1958.8.3.271

Laux, L., Glanzmann, P., Schaffner, P., & Spielberger, C. D. (1981). *Das State-Trait-Angstinventar (STAI).* Theoretische Grundlagen und Handanweisung. Beltz Test GmbH.

Tappen R, Barry C. Assessment of affect in advanced Alzheimer's disease: The Dementia Mood Picture Test. Journal of Housing for the Elderly. 1995;21(3):44–46. doi: 10.3928/0098-9134-19950301-09

Podsiadlo, D., & Richardson, S. (1991). The timed “Up & Go”: A test of basic functional mobility for frail elderly persons. *Journal of the American Geriatrics Society, 39*(2), 142–148. https://doi.org/10.1111/j.1532-5415.1991.tb01616.x

Rossiter-Fornoff, J. E., Wolf, S. L., Wolfson, L. I., & Buchner, D. M. (1995). A cross-sectional validation study of the FICSIT common data base static balance measures. *Journal of Gerontology: Medical Sciences, 50A*(6), M291–M297. https://doi.org/10.1093/gerona/50A.6.M291

Flansbjer, U. B., Holmbäck, A. M., Downham, D., Patten, C., & Lexell, J. (2005). Reliability of gait performance tests in men and women with hemiparesis after stroke. *Journal of Rehabilitation Medicine, 37*(2), 75–82. https://doi.org/10.1080/16501970410017215

Kristensen MT, Bloch ML, Jønsson LR, Jakobsen TL. Interrater reliability of the standardized Timed Up and Go Test when used in hospitalized and community-dwelling older individuals. Physiother Res Int. 2019 Apr;24(2):e1769. doi: 10.1002/pri.1769
