# Supplementary material for: Assessing the feasibility of interactions within a computer-generated virtual reality for people with dementia
Source: J Rehabil Assist Technol Eng. 2025 Nov 27;12:20556683251393992. doi: 10.1177/20556683251393992 (PMC12660644; doi:10.1177/20556683251393992)
Supplement: Supplemental Material - Assessing the feasibility of interactions within a computer-generated virtual reality for people with dementia [file sj-pdf-2-jrt-10.1177_20556683251393992.pdf]

<u>Feedback form after VR</u>

Indicate on a scale of 1-5 (1=strongly disagree; 5=strongly agree) whether you agree or disagree with the following statements:

		1	2	3	4	5
1	Did you find the VR tool enjoyable?	○	○	○	○	○
2	Would you use the VR tool again?	○	○	○	○	○
3	Would you like to try other VR tools?	○	○	○	○	○
4	Would you like to see more environments like this?	○	○	○	○	○
5	Would you like to spend more time viewing this environment?	○	○	○	○	○
6	Was this experience boring?	○	○	○	○	○
7	How real did the virtual reality feel to you?	○	○	○	○	○
8	Was this experience fascinating?	○	○	○	○	○
9	Was there too much going on?	○	○	○	○	○
10	Did you feel panicked while viewing this?	○	○	○	○	○
11	Did you feel confused or disoriented?	○	○	○	○	○
12	Was it easy for you to get used to the VR headset?	○	○	○	○	○
13	Did you find the VR headset too heavy?	○	○	○	○	○
14	Did the VR headset impair you?	○	○	○	○	○

15	Did you feel nauseous while viewing the VR?	○	○	○	○	○
16	Did you feel dizzy while viewing the VR?	○	○	○	○	○
17	Did you manage well with the controllers?	○	○	○	○	○
18	Could you easily move your head up, down, and to the sides?	○	○	○	○	○
